# Prognostic significance of cardiac magnetic resonance-based markers in patients with hypertrophic cardiomyopathy

**DOI:** 10.1007/s10554-021-02165-8

**Published:** 2021-02-08

**Authors:** Zsofia Dohy, Liliana Szabo, Attila Toth, Csilla Czimbalmos, Rebeka Horvath, Viktor Horvath, Ferenc Imre Suhai, Laszlo Geller, Bela Merkely, Hajnalka Vago

**Affiliations:** Heart and Vascular Center, Semmelweis University, 68 Varosmajor St, Budapest, 1122 Hungary

**Keywords:** Hypertrophic cardiomyopathy, Cardiac magnetic resonance, Prognosis, Left ventricular hypertrophy, Feature-tracking strain analysis, Myocardial fibrosis

## Abstract

The prognosis of patients with hypertrophic cardiomyopathy (HCM) varies greatly. Cardiac magnetic resonance (CMR) is the gold standard method for assessing left ventricular (LV) mass and volumes. Myocardial fibrosis can be noninvasively detected using CMR. Moreover, feature-tracking (FT) strain analysis provides information about LV deformation. We aimed to investigate the prognostic significance of standard CMR parameters, myocardial fibrosis, and LV strain parameters in HCM patients. We investigated 187 HCM patients who underwent CMR with late gadolinium enhancement and were followed up. LV mass (LVM) was evaluated with the exclusion and inclusion of the trabeculae and papillary muscles (TPM). Global LV strain parameters and mechanical dispersion (MD) were calculated. Myocardial fibrosis was quantified. The combined endpoint of our study was all-cause mortality, heart transplantation, malignant ventricular arrhythmias and appropriate implantable cardioverter defibrillator (ICD) therapy. The arrhythmia endpoint was malignant ventricular arrhythmias and appropriate ICD therapy. The LVM index (LVMi) was an independent CMR predictor of the combined endpoint independent of the quantification method (p < 0.01). The univariate predictors of the combined endpoint were LVMi, global longitudinal (GLS) and radial strain and longitudinal MD (MDL). The univariate predictors of arrhythmia events included LVMi and myocardial fibrosis. More pronounced LV hypertrophy was associated with impaired GLS and increased MDL. More extensive myocardial fibrosis correlated with impaired GLS (p < 0.001). LVMi was an independent CMR predictor of major events, and myocardial fibrosis predicted arrhythmia events in HCM patients. FT strain analysis provided additional information for risk stratification in HCM patients.

## Introduction

Hypertrophic cardiomyopathy (HCM) is a primary myocardial disease that is characterized by substantial heterogeneity in phenotypic expression, clinical course, and overall prognosis. HCM is one of the most common causes of sudden cardiac death (SCD) in young patients; another complication of HCM is progressive heart failure [1, 2]. The typical pathological features of HCM include myocyte disarray, small-vessel disease, and myocardial fibrosis, which usually have a patchy mid-myocardial distribution in the hypertrophic segments [3]. The structural abnormalities in HCM cause alterations in left ventricular (LV) mechanics [4]. The relation of the structural and mechanical characteristics to the clinical outcomes of patients with HCM has been partially explored.

According to the current European and American guidelines on HCM, the SCD risk stratification is based on age, family history of SCD, maximal LV wall thickness, left atrial diameter, LV outflow tract gradient, previous unexplained syncope and the occurrence of non-sustained ventricular tachycardia [5, 6], however, data in the literature suggest an additional prognostic role of the detection and quantification of myocardial fibrosis using cardiac magnetic resonance (CMR) examination [7, 8].

LV hypertrophy is usually measured using maximal end-diastolic wall thickness, and the diagnosis of HCM and SCD risk stratification are based on this parameter. However, LV mass (LVM) may more accurately describe hypertrophy. CMR-based quantification of LVM can be carried out with different evaluation methods. During conventional evaluation, the endocardial layer is detected along the inner border of the compact myocardium, resulting in the trabeculae and papillary muscle (TPM) being included in the ventricular cavity. Threshold-based methods define the endocardial surface based on the different signal intensities of blood and myocardium; thus, the TPM are measured as part of the ventricular mass [9]. There is no consensus on which evaluation method is more reliable in the case of HCM [10].

Strain analysis is a useful and reliable method for the assessment of global and regional myocardial function. Global LV strain parameters are considered a sensitive and early marker of LV dysfunction. The damage in different myocardium layers affects certain directions of deformation during LV contraction that can be detected using strain analysis even with normal ejection fraction (EF). Because of myocardial disarray and fibrosis, myocardial contraction in HCM is heterogeneous. Mechanical dispersion (MD) assessed by strain measurement reflects this heterogeneous contraction [11]. Strain analysis can help in the diagnosis and may guide the prognosis of patients with HCM; however, these findings are based on speckle tracking echocardiography [4, 12, 13]. Feature-tracking (FT) CMR has been validated for strain measurement using standard cine CMR images [14]. However, limited data are available on whether CMR-based strain analysis has incremental prognostic value in patients with HCM [15, 16].

Despite the advantages of the detailed evaluation of HCM patients, including strain analysis and myocardial fibrosis quantification, there is a lack of comprehensive studies with large study populations that integrate the prognostic significance of all of these data provided by CMR. Therefore, we conducted a study with the aim of investigating the prognostic significance of the following CMR parameters in one model: (1) different LV hypertrophy parameters such as LVM calculated using conventional and threshold-based methods and maximal end-diastolic wall thickness; (2) LV functional parameters such as ejection fraction (EF) and FT-CMR-based global LV strain values and MD; and (3) amount of myocardial fibrosis.

## Methods

### Study population

**Fig. 1:**
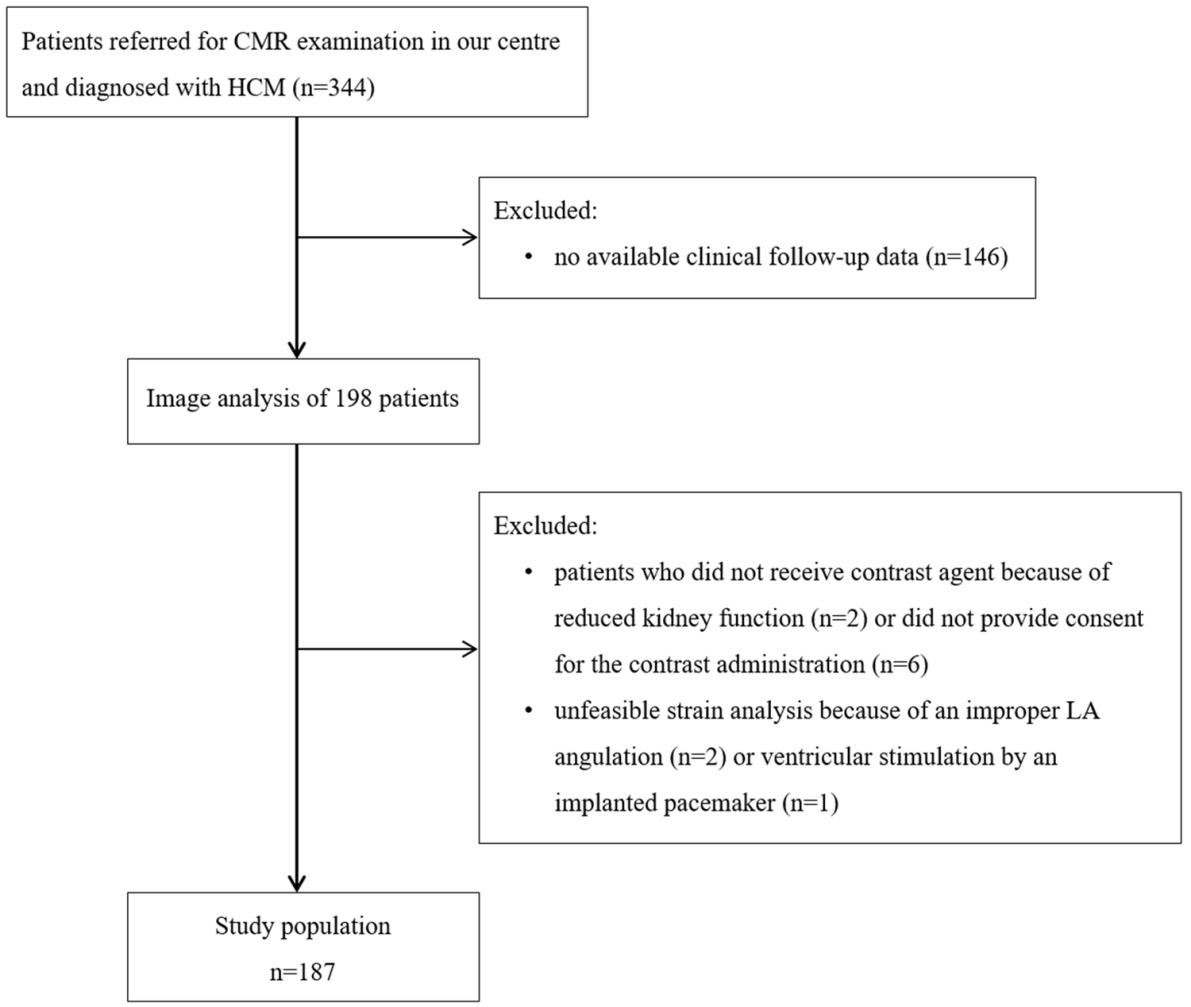
Study flow chart

We enrolled consecutive patients who underwent CMR examination in our tertiary referral centre between January 2009 and March 2019. The patients were followed up in this retrospective longitudinal observational study. Our inclusion criteria were the unequivocal diagnosis of HCM and lack of confounding comorbidities, such as untreated hypertension, significant aortic stenosis, previous myocardial infarction and prior surgical myectomy or percutaneous transluminal septal myocardial ablation. We excluded patients if there were no available clinical follow-up data, they did not receive contrast agent, or if the strain analysis was not properly feasible (Fig. 1).

Informed consent was obtained from each patient. Ethical approval was obtained from the Hungarian National Institute of Pharmacy and Nutrition (OGYEI/29,174–4/2019), and this study was performed in accordance with the ethical standards in the 1964 Declaration of Helsinki and its later amendments.

### CMR protocol

CMR examinations were conducted with a 1.5 T magnetic resonance (MR) scanner (Achieva, Philips Medical Systems) using a 5-channel cardiac coil*.* Retrospectively gated balanced steady-state free precession (bSSFP) cine images were acquired in 2-chamber, 4-chamber and LV outflow tract views. Additionally, short-axis (SA) images with full coverage of the LV were obtained. A bolus of gadobutrol (0.15 mmol/kg) was injected at a rate of 2–3 ml/s through an antecubital intravenous line. Late gadolinium enhancement (LGE) images were acquired using a segmented inversion recovery sequence with additional phase-sensitive reconstructions in the same views used for cine images 10–20 min after contrast administration.

### Image analysis

**Fig. 2:**
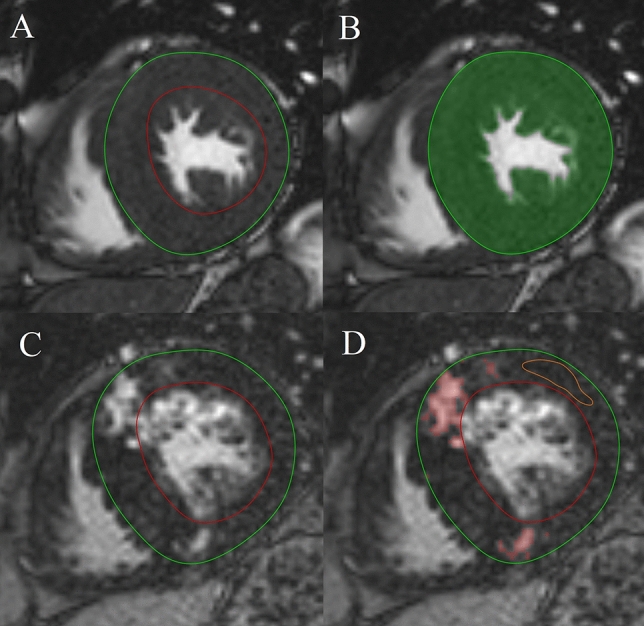
CMR images of a patient with septal HCM. **a**, **b** bSSFP cine short-axis image in the end-diastolic phase. Measurement of LV mass with conventional (**a**) and threshold-based (**b**) methods. **c**, **d** Delayed contrast enhancement images in the short-axis plane. **d** Quantification of myocardial fibrosis at 5 SD

CMR data were analysed using Medis Suite 3.1 software (Medis Medical Imaging Software, Leiden, The Netherlands). The left ventricular ejection fraction (LVEF), volumes and LVM were quantified using conventional and threshold-based methods. During conventional contouring, the endo- and epicardial layers were manually traced along the inner and outer borders of the compact myocardium. For TPM quantification, a thresholding algorithm was used (MassK 7.8, Medis, Leiden, The Netherlands), which distinguishes between blood and myocardium based on their different signal intensities (Fig. 2). We used the same end-diastolic and end-systolic phases for the threshold-based quantification. LV volumes, LVM and TPM were standardized to the body surface area (BSA). Maximal end-diastolic wall thickness measurements were taken in an SA slice perpendicular to the myocardial centre line, excluding trabeculation. On LGE images, myocardial fibrosis was quantified at a grey-scale threshold of 5 standard deviations (SDs) above the mean signal intensity for normal myocardium (Fig. 2). LV strain analysis was obtained with the feature-tracking application of the MedisSuite: QStrain module. Endocardial contour detection was manually performed on long-axis (LA) and SA cine images in end-systolic and end-diastolic phases. Global longitudinal (GLS), circumferential (GCS) and radial (GRS) LV strain parameters were measured. For global dyssynchrony measurement, MD was assessed, which was defined as the SD of the time-to-peak circumferential (MDC) and longitudinal (MDL) strain of the LV segments expressed as percentage of the cardiac cycle.

### Endpoint and clinical follow-up

The clinical follow-up was based on the medical records and the National Health Insurance Fund of Hungary record database, which includes up-to-date information on the date of death. A combined endpoint and an arrhythmia endpoint were analysed. The combined endpoint included all-cause mortality, heart transplantation, and malignant ventricular arrhythmia or appropriate implantable cardioverter defibrillator (ICD) therapy. The arrhythmia endpoint included malignant ventricular arrhythmia and appropriate ICD therapy.

### Statistical analysis

Continuous data are expressed as the mean ± SD. The normal distribution of data was investigated with the Kolmogorov–Smirnov test. The characteristics of groups were compared with an independent t-test or Mann–Whitney test, as appropriate. The correlation between continuous variables was calculated with Spearman’s correlation analysis. The prognostic value of CMR parameters was assessed with univariate and multivariate Cox proportional hazard regression analyses with the enter selection method. Variables with p < 0.05 in univariate analyses were candidates for multivariate analyses. Multicollinearity was measured with the variance inflation factor (VIF). Highly correlated predictors (VIF > 2.5) were removed from the multivariate model. Receiver operating characteristic (ROC) curve analysis was performed to identify optimal cut-off values. Univariate associations of time variables with major events were visualized using Kaplan–Meier curves and compared by the log-rank test. Differences were considered statistically significant when p < 0.05. All analyses were performed by using MedCalc software (version 17.9.5).

## Results

### Patient characteristics

The demographic and CMR characteristics of the study population are summarized in Table 1. The patients had the following symptoms: syncope (19%), chest pain (41%), dyspnoea (39%), and palpitation (36%). Three patients were examined after aborted SCD. Based on echocardiography and electrocardiography (ECG) findings, HCM was the referral diagnosis in 96% of the cases, while in the remaining 4% of the patients, the referral diagnosis was amyloidosis, left ventricular noncompaction, arrhythmogenic cardiomyopathy, anomalous origin of a coronary artery, previous myocardial infarction or undefined structural abnormality. Echocardiography described obstructive HCM in 34% of the cases.

**Table 1:** Demographic and CMR characteristics of the study population. Comparison of the parameters of patients with and without combined or arrhythmia endpoints

	Combined endpoint			Arrhythmia endpoint		
	Yes	No	p	Yes	No	p
Number of patients	34	153		12	168	
Male	13 (38%)	86 (56%)	0.06	5 (42%)	92 (55%)	0.38
Age (y)	47.8 ± 20.9	46.8 ± 17.9	0.52	36.0 ± 22.0	47.4 ± 17.9	0.09
BSA (m^2^)	1.81 ± 0.26	1.91 ± 0.25	0.06	1.70 ± 0.37	1.90 ± 0.25	0.07
LVEF (%)	62.4 ± 6.9	62.9 ± 7.7	0.74	60.2 ± 8.0	62.9 ± 7.6	0.26
LVESVi (ml/m^2^)	34.9 ± 13.4	33.2 ± 10.8	0.51	38.2 ± 16.2	33.3 ± 11.0	0.24
LVEDVi (ml/m^2^)	91.7 ± 26.4	88.1 ± 17.0	0.65	93.8 ± 28.2	88.4 ± 18.2	0.65
LVSVi (ml/m^2^)	56.6 ± 15.5	55.4 ± 10.9	0.95	55.2 ± 14.6	55.5 ± 11.7	0.67
LVMi_conv_ (g/m^2^)	114.9 ± 52.1	88.0 ± 31.2	< 0.001	126.2 ± 56.5	90.4 ± 35.0	< 0.01
LVMi_TB_ (g/m^2^)	142.2 ± 67.5	113.0 ± 37.4	< 0.01	160.8 ± 75.2	115.5 ± 42.1	< 0.01
TPMi (g/m^2^)	29.0 ± 15.2	24.9 ± 8.4	0.17	34.0 ± 19.7	25.1 ± 9.0	< 0.05
Maximal wall thickness (mm)	22.2 ± 5.7	20.6 ± 5.7	0.14	23.0 ± 6.0	20.9 ± 5.7	0.20
Myocardial fibrosis (g)	20.9 ± 18.6	16.6 ± 21.4	< 0.05	29.3 ± 22.9	16.4 ± 21.0	< 0.05
Myocardial fibrosis (%)	9.8 ± 7.4	8.4 ± 8.9	0.12	13.1 ± 8.7	8.2 ± 8.7	< 0.05
GLS (%)	− 21.2 ± 6.2	− 22.9 ± 5.4	0.20	− 20.6 ± 6.9	− 22.7 ± 5.4	0.27
GCS (%)	− 40.3 ± 8.6	− 40.2 ± 7.5	0.90	− 39.1 ± 9.0	− 40.0 ± 7.5	0.68
GRS (%)	76.6 ± 22.0	83.4 ± 22.5	0.11	74.8 ± 21.1	82.4 ± 22.6	0.26
MDL (%)	17.7 ± 4.6	16.4 ± 5.2	0.17	17.7 ± 5.7	16.5 ± 5.1	0.44
MDC (%)	8.5 ± 4.7	7.1 ± 3.8	0.10	9.3 ± 5.0	7.2 ± 3.8	0.16

### CMR characteristics

**Fig. 3:**
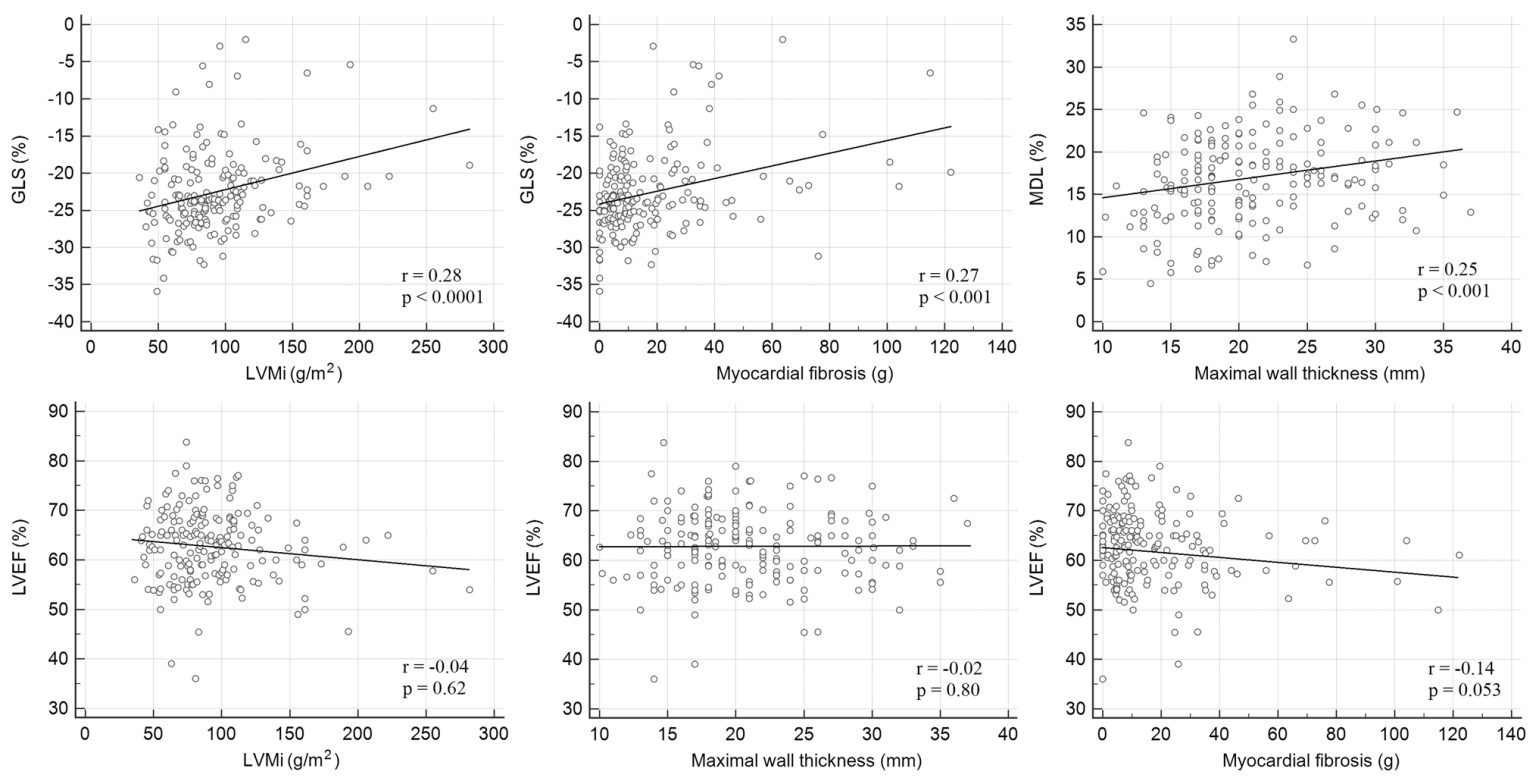
Correlation between LV functional parameters and LV hypertrophy and myocardial fibrosis (Spearman’s correlation)

The majority of the study population (147 patients) had a normal LVEF (57–77%), two patients had a supra-normal LVEF (> 77%), 36 patients had a mildly reduced LVEF (41–56%), and two patients had a moderately reduced LVEF (30–40%). The most common form of HCM was asymmetric hypertrophy with a septal or an anterior distribution, which was found in 161 patients (81.5%). There were 27 (13.5%) patients with apical HCM, 7 (3.5%) patients with concentric HCM and three (1.5%) patients with midventricular HCM. Myocardial fibrosis was detected in 90.6% of patients. More extensive myocardial fibrosis was associated with a higher LVM index (LVMi) (p < 0.0001, r = 0.495) and higher maximal end-diastolic wall thickness (p < 0.0001, r = 0.44). Impaired GLS correlated with a higher LVMi and more extensive myocardial fibrosis. Higher maximal end-diastolic wall thickness correlated with higher MDL, referring to more pronounced global LV dyssynchrony. LVEF did not correlate with LV hypertrophy or with the amount of myocardial fibrosis (Fig. 3).

### Clinical outcome

During the follow-up (3.8 ± 2.4 years), 20 patients died, and the cause of death was known in 11 patients. One patient died because of brain cancer, cardiovascular death was obvious in 10 cases, and one of them had SCD. In the case of 9 patients, the cause of death was unknown, and these patients were not included in the statistical analyses regarding arrhythmia events. Six patients underwent heart transplantation. One patient had aborted SCD during the follow-up period. ICD implantation occurred in 52 patients (48 as primary prevention), appropriate ICD therapy was detected in 9 patients (6 DC shock, 3 antitachycardia pacing), and one patient had electrical storm. The three patients who were examined after aborted SCD had appropriate ICD therapy during the follow-up.

### The prognostic value of CMR

The patients who reached the combined endpoint had higher LVMi both with the conventional (LVMi_conv_) and threshold-based (LVMi_TB_) evaluation methods. The patients with arrhythmia events had higher LVMi_conv_ and LVMi_TB_, higher TPM index (TPMi) and more extended myocardial fibrosis (Table 1).

In the apical HCM group, the endpoint of our study was detected in only one patient who had heart transplantation; however, statistically, there was no difference in the prognosis of the different morphological types of HCM.

LVMi_conv,_ LVMi_TB_, GLS, GRS and MDL were significant univariate predictors of the combined endpoint. In the multivariate models, LVMi was an independent predictor of the combined endpoint (p < 0.01). We investigated the prognostic factors of arrhythmia events, and we found that LV end-systolic volume index (ESVi), LVEF, LVMi_conv,_ LVMi_TB,_ TPMi and myocardial fibrosis were significant univariate predictors of arrhythmia events (Table 2).

**Table 2:** Predictors of the combined and arrhythmia endpoints assessed with univariate and multivariate Cox proportional hazard regression analyses

	Univariate analysis		Multivariate analysis with LVMi_Conv_		Multivariate analysis with LVMi_TB_	
	p	HR [95% CI]	p	HR [95% CI]	p	HR [95% CI]
**Combined endpoint**						
Age	0.21	1.01 [0.99 to 1.03]				
Female gender	0.06	1.93 [0.97 to 3.87]				
LVEDVi	0.56	1.01 [0.99 to 1.02]				
LVESVi	0.13	1.02 [0.99 to 1.05]				
LVSVi	0.57	0.99 [0.96 to 1.02]				
LVEF	0.051	0.95 [0.91 to 1.00]				
LVMi_Conv_	0.002	1.01 [1.003 to 1.02]	0.011	1.01 [1.00 to 1.02]		
LVMi_TB_	0.005	1.01 [1.002 to 1.01]			0.02	1.01 [1.00 to 1.01]
TPMi	0.07	1.02 [1.00 to 1.04]				
Maximal end-diastolic wall thickness	0.90	1.004 [0.95 to 1.06]				
Myocardial fibrosis (%)	0.42	1.01 [0.98 to 1.05]				
Myocardial fibrosis (g)	0.51	1.005 [0.99 to 1.02]				
GLS	0.02	1.08 [1.01 to 1.15]	0.27	1.04 [0.97 to 1.12]	0.26	1.04 [0.97 to 1.12]
GCS	0.81	1.01 [1.01 to 1.05]				
GRS	0.048	0.98 [0.97 to 0.99]				
MDL	0.048	1.07 [1.00 to 1.14]	0.12	1.06 [0.99 to 1.13]	0.13	1.06 [0.98 to 1.13]
MDC	0.06	1.08 [0.99 to 1.17]				
**Arrhythmia endpoint**						
Age	0.19	0.98 [0.94 to 1.01]				
Female gender	0.34	1.76 [0.55 to 5.59]				
LVEDVi	0.54	1.01 [0.98 to 1.04]				
LVESVi	0.08	1.04 [1.00 to 1.08]				
LVSVi	0.39	0.98 [0.93 to 1.03]				
LVEF	0.03	0.91 [0.84 to 0.99]	0.15	0.93 [0.84 to 1.03]	0.13	0.92 [0.83 to 1.06]
LVMi_Conv_	0.01	1.01 [1.00 to 1.02]	0.28	1.01 [0.99 to 1.03]		
LVMi_TB_	0.009	1.01 [1.00 to 1.02]			0.21	1.01 [0.99 to 1.03]
TPMi	0.02	1.03 [1.00 to 1.06]	0.71	0.99 [0.92 to 1.06]	0.50	0.97 [0.89 to 1.06]
Maximal end-diastolic wall thickness	0.76	1.02 [0.92 to 1.12]				
Myocardial fibrosis (%)	0.03	1.05 [1.01 to 1.09]	0.14	1.03 [0.99 to 1.08]	0.15	1.03 [0.99 to 1.08]
Myocardial fibrosis (g)	0.07	1.02 [1.00 to 1.04]				
GLS	0.053	1.11 [1.00 to 1.22]				
GCS	0.58	1.02 [0.95 to 1.10]				
GRS	0.15	0.98 [0.95 to 1.01]				
MDL	0.22	1.07 [0.96 to 1.19]				
MDC	0.09	1.12 [0.98 to 1.27]				

In the analysis of the predictors of the combined endpoint, LVMiconv, LVMiTB, GLS, GRS and MDL were significant in the univariate analyses. Multicollinearity was measured with the variance inflation factor (VIF), and GLS and GRS were highly correlated predictors (VIF > 2.5); therefore, GRS was removed from the model. LVMi_conv_ and LVMi_TB_ were significant predictors of the combined endpoint in the multivariate analysis. In the analysis of the predictors of the arrhythmia endpoint, the significant univariate predictors were LVESVi, LVEF, LVMi, TPMi and myocardial fibrosis

**Fig. 4:**
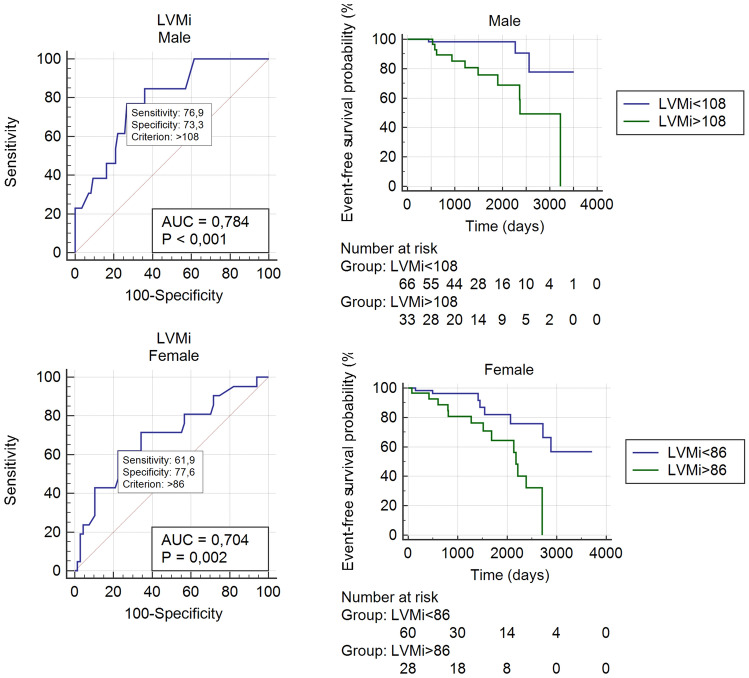
LVMi cut-off for males and females with ROC analysis regarding major events. Event-free survival of patients divided by LVMi cut-off (Kaplan–Meier curves)

Using ROC analysis, we calculated different LVMi cut-offs regarding the combined endpoint: the LVMi cut-off for males with the conventional evaluation method was 108 g/m^2^, and it was 128 g/m^2^ with the threshold-based method. The LVMi cut-off for females with the conventional evaluation method was 86 g/m^2^, and it was 107 g/m^2^ with the threshold-based method. The patients with an LVMi less than the cut-off value had significantly better prognosis (Fig. 4).

## Discussion

The main findings of our single-centre study with a relatively large HCM population are as follows:

1. LVMi was an independent CMR predictor of major events (mortality, heart transplantation, malignant ventricular arrhythmia or appropriate ICD therapy) independent of the LVMi quantification method.
2. The univariate predictors of major events were LVMi, GLS, GRS and MDL. The univariate predictors of arrhythmia events were LVESVi, LVEF, LVMi, TPMi and myocardial fibrosis.
3. More pronounced LV hypertrophy was associated with impaired GLS and increased MDL. More extended myocardial fibrosis correlated with impaired GLS. However, LVEF showed no correlation with the degree of LV hypertrophy or with the extent of myocardial fibrosis.

The primary role for CMR in patients with HCM is the clarification of the diagnosis. The prognostic significance of CMR has been under discussion in this patient population; therefore, the current guidelines on HCM do not contain CMR examination in the risk stratification [6], or recommend that CMR be considered in selected patients with HCM for whom risk remains borderline after documentation of conventional risk factors [5]. Several studies have demonstrated that patients with more extensive myocardial fibrosis have a higher risk for malignant ventricular arrhythmias [7, 8, 17, 18]. Myocardial fibrosis in HCM consists of varying degrees of both replacement fibrosis and interstitial fibrosis. In our study, both diffuse and definite contrast enhancement were considered indications of myocardial fibrosis, which explains the high proportion (90.6%) of patients with myocardial fibrosis. Based on a previous study, the 5SD quantification method showed the strongest correlation with the histopathological assessment of total fibrosis (the sum of replacement and interstitial fibrosis) [19]. In our prognostic model, the extent of myocardial fibrosis (assessed with 5SD) was analysed. Our results are consistent with the supposition of the prognostic role of myocardial fibrosis; in our patient population, myocardial fibrosis was a univariate predictor of malignant ventricular arrhythmias.

Conventionally, maximal end-diastolic wall thickness is used to describe LV hypertrophy and to estimate SCD risk in HCM [20–22]. However, data in the literature regarding the prognostic significance of maximal wall thickness have been controversial [23]. LVM is a more robust measure of the total burden of LV hypertrophy than a single measurement of the maximal wall thickness. Spiewak et al. stated that the maximal wall thickness does not reflect the degree of LV hypertrophy in patients with HCM, as patients with the same wall thickness may have substantial differences in LVM [24]. Olovitto et al. found that markedly increased LVMi was more sensitive in predicting outcome, whereas maximal end-diastolic wall thickness > 30 mm was more specific in HCM patients [25]. CMR examination provides the most accurate and reproducible information about LVM, as CMR-based LVM measurements are free of cardiac geometric assumptions [26]. There are different evaluation methods of LVM that either include TPM or exclude it from the mass. We found that higher LVMi predicted poor clinical outcome independent of the evaluation method; nevertheless, the LVMi cut-off values regarding major events depended on the evaluation method and sex. In our patient population, maximal end-diastolic wall thickness was not a predictor of major events.

Myocardial strain analyses provide accurate information about global and regional LV function. Strain by speckle tracking echocardiography has been increasingly applied as a sensitive and early marker of LV dysfunction in different cardiomyopathies. Previous studies have demonstrated the relation of echocardiography-based strain parameters to structural alterations and clinical outcomes in patients with HCM [4, 12, 27]. Hinojar et al. investigated the prognostic implication of global strain parameters assessed with FT-CMR in 74 patients with HCM. They found that impaired global LV strain values were associated with all-cause mortality and heart failure events [15]. MD was not investigated in their study; however, a previous study demonstrated the prognostic role of MD evaluated with FT-CMR in patients after ST-segment elevation myocardial infarction [28]. In our study, GLS, GRS and MDL were univariate predictors of major events. In patients with more pronounced LV hypertrophy, we found increased global LV dyssynchrony and impaired longitudinal contraction, while LVEF did not correlate with the degree of hypertrophy. These results suggested that the FT strain analysis provides important additional information for the detection of LV dysfunction and for risk stratification in HCM patients.

## Study limitations

The limitations of our study are that it was a single-centre study, which might limit the generalizability of our conclusions. Although our study was designed to represent a real-world population, due to the retrospective nature of the study, limited clinical data were available for some patients; therefore, these patients were excluded from the analyses. Myocardial T1 and T2 mapping and myocardial extracellular volume evaluation were not available. In the vast majority of the patients, no genetic testing was performed.

## Conclusion

Our results show that LVMi is an independent CMR predictor of major events and that myocardial fibrosis predicts arrhythmia events in HCM patients. Furthermore, FT strain analysis provides additional information for the detection of LV dysfunction and for risk stratification in HCM patients.
